# Ponatinib exerts anti-angiogenic effects in the zebrafish and human umbilical vein endothelial cells via blocking VEGFR signaling pathway

**DOI:** 10.18632/oncotarget.24110

**Published:** 2018-01-10

**Authors:** Nana Ai, Cheong-Meng Chong, Weiting Chen, Zhe Hu, Huanxing Su, Guokai Chen, Queenie Wing Lei Wong, Wei Ge

**Affiliations:** ^1^ Centre of Reproduction, Development and Aging (CRDA), Faculty of Health Sciences, University of Macau, Macau, China; ^2^ Institute of Chinese Medicinal Sciences (ICMS), University of Macau, Macau, China

**Keywords:** ponatinib, angiogenesis, eNOS, MAPK, HUVEC

## Abstract

Angiogenesis is a hallmark for cancer development because it is essential for cancer growth and provides the route for cancer cell migration (metastasis). Understanding the mechanism of angiogenesis and developing drugs that target the process has therefore been a major focus for research on cancer therapy. In this study, we screened 114 FDA-approved anti-cancer drugs for their effects on angiogenesis in the zebrafish. Among those with positive effects, we chose to focus on Ponatinib (AP24534; Iclusig^®^) for further investigation. Ponatinib is an inhibitor of the tyrosine kinase BCR-ABL in chronic myeloid leukemia (CML), and its clinical trial has been approved by FDA for the treatment of the disease. In recent clinical trials, however, some side effects have been reported for Ponatinib, mostly on blood vessel disorders, raising the possibility that this drug may influence angiogenesis. In this study, we demonstrated that Ponatinib was able to suppress the formation of intersegmental vessels (ISV) and subintestinal vessels (SIV) in the zebrafish larvae. The anti-angiogenic effect of Ponatinib was further validated by other bioassays in human umbilical vein endothelial cells (HUVECs), including cell proliferation and migration, tube formation, and wound healing. Further experiments showed that Ponatinib inhibited VEGF-induced VEGFR2 phosphorylation and its downstream signaling pathways including Akt/eNOS/NO pathway and MAPK pathways (ERK and p38MAPK). Taken together, these results suggest that inhibition of VEGF signaling at its receptor level and downstream pathways may likely be responsible for the antiangiogenic activity of Ponatinib.

## INTRODUCTION

Angiogenesis is the process of new blood vessel formation and maturation from pre-existing vessels, which involves a series of cellular activities or processes including proliferation and migration of endothelial cells, vessel formation and vascular maturation [1]. Angiogenesis is stimulated by various angiogenic factors such as vascular endothelial growth factor (VEGF), fibroblast growth factor (FGF) family, and angiopoietins. Dysregulation of angiogenesis is implicated in several diseases, including cancer, chronic inflammation, and wound healing [2–5]. Tumor angiogenesis is considered a main hallmark in cancer progression. It provides blood to tumors for supplying nutrients and oxygen and removing waste products, therefore playing a critical role in tumor cell growth and survival [6]. Tumors and nearby normal cells in tumor microenvironment can secret proangiogenic factors to stimulate new blood vessel formation for blood supply [7]. In addition, cancer cells can invade nearby tissues, move throughout the body, and establish new colonies via new blood vessel formation [6, 8]. Recently, more and more drug candidates have been shown to suppress cancer growth through inhibiting angiogenesis [9, 10]. Therefore, targeting angiogenesis-related mechanisms is considered an effective approach to slow down or halt the progression of cancers.

In recent years, increasing studies have been performed in the zebrafish model for phenotype-based chemical screening to identify small molecules for drug development [11]. In addition to discovery of novel compounds, repurposing screen in the zebrafish is also useful for identifying new applications of existing drugs. Through such screening, some clinically approved drugs, such as cyclooxygenase inhibitors and glucocorticoid flurandrenolide, have been identified as potent suppressors of leukaemia-like phenotype and long QT syndrome, respectively [12, 13]. Since these drugs have been well characterized on pharmacokinetics and safety and are being used in humans, the new applications would abbreviate the process of clinical investigation [14]. Zebrafish has been demonstrated to be an excellent model for studying angiogenesis and screening for potential anti-angiogenic drugs because its process of angiogenesis is similar to that in other vertebrates [15–17]. Zebrafish angiogenesis involves differentiation of hemangioblasts from mesoderm, which subsequently differentiate into angioblasts and endothelial cells [18]. During early zebrafish embryogenesis, some developing vessels such as intersegmental vessels (ISVs) and subintestinal vessels (SIVs) are relatively simple [19, 20], and their formation is often used as markers for evaluating anti-angiogenic compounds after primary screening [21, 22]. Compared with other animal models, the advantages of zebrafish include easy experimentation, convenient drug administration, and amenability to *in vivo* manipulation [23, 24]. In addition, the small size, high fertility rate, fast embryonic development, and easy analysis for vascular development make zebrafish a convenient model for *in vivo* high-throughput experiments [25].

Using zebrafish as the model, we recently screened 114 FDA-approved anti-cancer drugs for potential anti-angiogenic effects. Twelve drugs were found to exhibit anti-angiogenic activities, including Ponatinib (Supplementary Table 1). Ponatinib (AP24534; Iclusig^®^) was originally developed as a potent inhibitor of tyrosine kinase BCR-ABL, a fusion gene product carried by the Philadelphia chromosome (Ph^+^ ALL), and its mutant forms especially T315I [26, 27]. It was approved by FDA in 2012 for treating the patients of chronic myeloid leukemia (CML), in particular those with Ph^+^ ALL who are resistant to the therapies with the first- and second-line tyrosine kinase inhibitors imatinib, dasatinib and nilotinib [28, 29]. In addition, Ponatinib has also been reported to be effective in suppressing cell growth of other types of cancers such as acute myeloid leukemia (AML) [30], chronic eosinophilic leukemia [31], non-small cell lung cancers [32] and endometrial cancers [33]. However, FDA has recently reported a high frequency of blood clotting and narrowing of blood vessels in the clinical trial for Ponatinib [34]. These side effects on vascular system raise an interesting question about potential effects of Ponatinib on the vascular system and its action mechanisms. In this study, we demonstrated that Ponatinib was able to suppress the formation of ISVs and SIVs in the zebrafish larvae. We further validated the effects of Ponatinib in a series of angiogenic bioassays using human umbilical vein endothelial cells (HUVECs). We also investigated the potential signaling pathways that might mediate the anti-angiogenic activity of Ponatinib.

## RESULTS

### Ponatinib suppressed angiogenesis in the zebrafish

**Figure 1:**
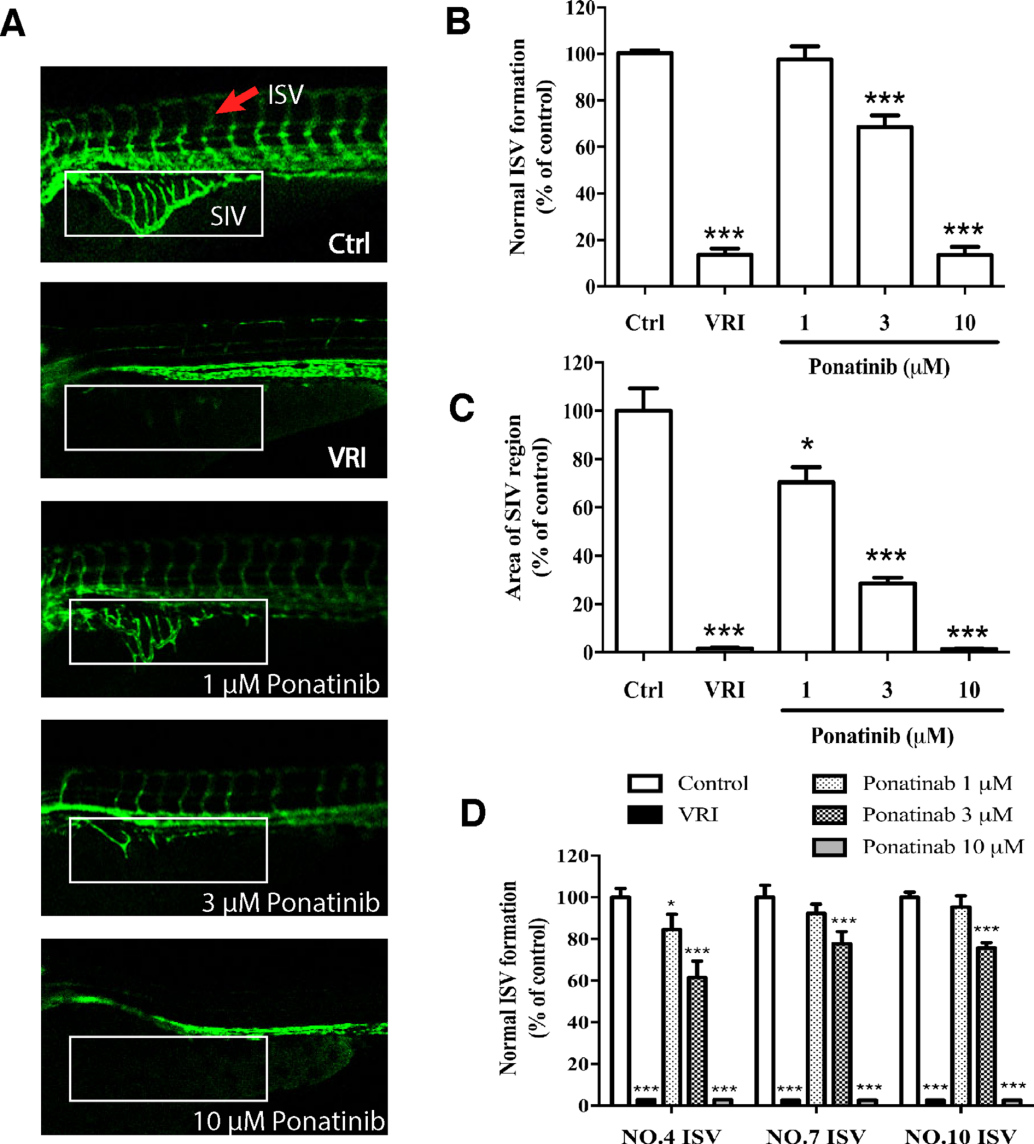
Effects of Ponatinib on vascular development of zebrafish larvae (**A**) Fluorescent images of ISV and SIV in the zebrafish embryos incubated with 0.1% DMSO (Ctrl: vehicle-control), VRI (5 μM), and Ponatinib (1, 3 and 10 μM) for 48 h. VRI was used as the positive control. Red arrow: normal ISV. (**B**) Number of normal ISV with full-length formation. (**C**) Quantification of SIV area as a percentage of the control group. The total SIV area was determined by using the NIH image J program. (**D**) Formation of selected ISV (NO. 4, 7, and 10). All values are presented as means ± SD (*n* = 12). ^*^P < 0.05 vs. control group, and ^***^P < 0.005 vs. control group.

In order to assess the *in vivo* effects of Ponatinib on vascular development, a transgenic zebrafish line *Tg(fli1a:EGFP)*, which expresses EGFP specifically in the vascular vessels, was used to monitor the formation of blood vessels, especially the ISVs and SIVs. The drug VRI, a known anti-angiogenic drug, was used as the positive control. The ISV formation was quantified by the number of normal vessels. As shown in Figure 1A and 1B, treatment of the embryos with Ponatinib at 1, 3 and 10 μM for 48 h suppressed normal ISV formation in a concentration-dependent manner (97%, 68%, and 13%, respectively). The maximum effect of Ponatinib was similar to that of VRI (13%). Consistently, Ponatinib also suppressed the formation of SIVs, and its effect was significant at 1 μM. At 10 μM, Ponatinib fully blocked the formation of SIVs, again similar to that of VRI (Figure 1A and 1C). In addition, the lengths of the 4th, 7th and 10th vessels of the ISV were quantified and shown to be significantly reduced by Ponatinib in all the embryos, which was similar to VRI (Figure 1D).

### Ponatinib attenuated viability and proliferation of HUVECs

**Figure 2:**
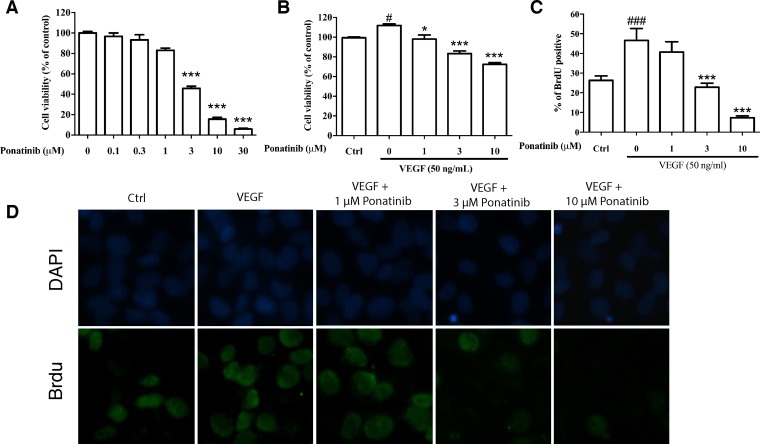
Effects of Ponatinib on viability and proliferation of HUVECs (**A**) Effect of Ponatinib on basal viability of HUVECs. The cells were treated with Ponatinib at indicated concentrations or 0.1% DMSO (vehicle control) for 24 h. (**B**) Effect of Ponatinib on cell viability of VEGF-treated HUVECs. The cells were treated with VEGF (50 ng/mL) and Ponatinib (1, 3 and 10 μM) for 24 h. Cell viability was measured using the MTT assay. (**C**) Effect of Ponatinib on basal and VEGF-induced proliferation of HUVECs. The cells were exposed to VEGF (50 ng/mL) and Ponatinib (1, 3 and 10 μM) in the presence of BrdU for 24 h. After immunostaining, the BrdU-positive cells were counted and presented as the percentage of total cells. (**D**) Representative images of BrdU-positive cells with DAPI staining the nuclei. Values are presented as means ± SD (*n* = 3). ^#^*P* < 0.05 and ^###^*P* < 0.005 vs. control group; ^*^P < 0.05 and ^***^P < 0.005 vs. VEGF-treated group.

To evaluate the effects of Ponatinib on cell viability and proliferation, the HUVECs were incubated with various concentrations of Ponatinib for 24 h and cell viability was assessed by MTT assay. As shown in Figure 2A, Ponatinib alone at 0.1, 0.3 and 1 μM did not show any effect on the viability of HUVECs, whereas higher doses of Ponatinib (3, 10 and 30 μM) showed significant effects in a concentration-dependent manner with ED_50_ being 5.3 μM. We also tested this effect in the presence of VEGF. Treatment of the HUVECs with 50 ng/mL VEGF increased cell viability (112%), and co-treatment with Ponatinib (1, 3 and 10 μM) attenuated the basal and VEGF-induced cell viability in a concentration-dependent manner (98%, 83% and 72%, respectively, compared to the control). Interestingly, in the presence of VEGF, Ponatinib showed much less effect on cell viability (Figure 2B). We further tested if Ponatinib could affect the VEGF-induced proliferation of HUVECs. Cell proliferation was measured by BrdU assay. VEGF significantly increased BrdU-positive cells (47%), compared with the control group (26%); however, co-treatment with Ponatinib (1, 3 and 10 μM) suppressed basal and VEGF induced HUVEC proliferation (Figure 2C and 2D).

### Ponatinib inhibited VEGF-induced tube formation of HUVECs

**Figure 3:**
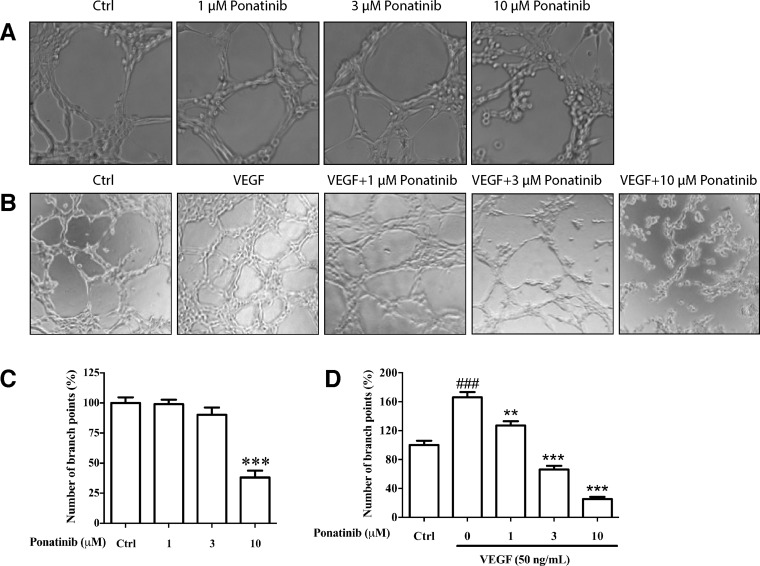
Effects of Ponatinib on basal and VEGF-induced tube formation in HUVECs (**A**) Representative images of the cells treated with Ponatinib (1, 3 and 10 μM) alone or (**B**) in combination with VEGF (50 ng/mL) for 7–8 h. (**C**–**D**) The branch points were counted and presented as the percentage of the control group. Values are presented as means ± SD (*n* = 3). ^###^*P* < 0.005 vs. control group; ^**^P < 0.01 and ^***^P < 0.005 vs. VEGF-treated group.

To test if Ponatinib has any effect on tube formation by cultured HUVECs, the cells were incubated on Matrigel with or without VEGF in the presence or absence of Ponatinib. Within 7-8 h of incubation, the HUVECs rearranged in an organized manner to form tubes in culture (Figure 3). Ponatinib alone had no effect on basal tube formation at low doses (1 and 3 μM) and the effect became significant at 10 μM (Figure 3A and 3C). Treatment with VEGF alone significantly increased the level of tube formation. In the presence of Ponatinib, the VEGF-induced tube formation was significantly reduced. At low dose of Ponatinib (1 μM), the number of branching points was significantly less as compared with the VEGF-treated group (Figure 3B and 3D).

### Ponatinib suppressed VEGF-induced migration of HUVECs

**Figure 4:**
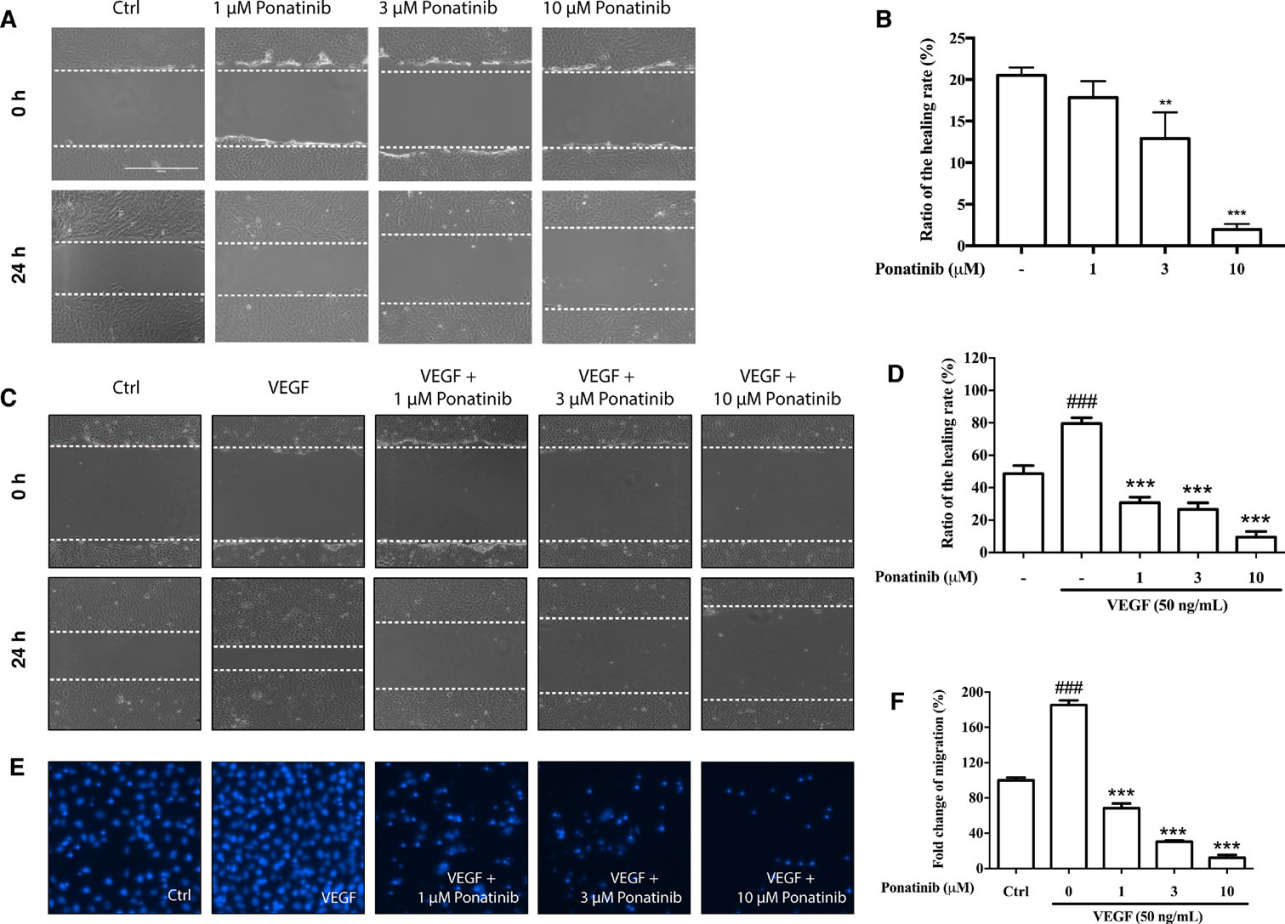
Effects of Ponatinib on cell migration of HUVECs (**A** and **C**) Representative images of wound healing assay. The dashed lines indicate wound edges. After scratching, the HUVECs were treated with VEGF (50 ng/mL) in the absence or presence of Ponatinib (1, 3 and 10 μM) for 24 h. (**B** and **D**) Quantification of the healing rate. The healing or closure rate was expressed as a ratio of the migration distance compared with the distance immediately after scratching. Values are presented as means ± SD (*n* = 3). ^###^*P* < 0.005 vs. control group; ^***^P < 0.005 vs. VEGF-treated group. (**E**) Representative images of transwell migration assay. HUVECs were pretreated with Ponatinb at the indicated concentration or 0.1% DMSO (vehicle control) in the upper compartment of the transwell insert. The lower chamber contained medium with or without VEGF (50 ng/mL), which serves as a chemo-attractant. After 24 h, cells remaining on the upper membrane were scraped off, and cells on the lower membrane surface were stained with DAPI for counting and quantification. (**F**) Quantification of migrated cells across the membrane. Values are presented as means ± SD (*n* = 3). ^###^*P* < 0.005 vs. control group; ^***^P < 0.005 vs. VEGF-treated group.

In the wound healing assay, Ponatinib at 1, 3, and 10 μM was able to reduce the basal and VEGF-induced migration of HUVECs after 24 h in a concentration-dependent manner (30%, 26% and 9%, respectively) (Figure 4A–4D). We also used transwell migration assay to measure the migratory response of HUVECs to Ponatinib. After drug treatment, the number of cells migrating across pores in 24 h was determined by counting stained cells on transwell filters. Ponatinib significantly suppressed the VEGF-induced cell migration across the transwell membrane (Figure 4E) in a concentration-dependent manner (68%, 30% and 12%, respectively, at 1, 3 and 10 μM) (Figure 4F).

### Ponatinib attenuated VEGF-induced NO generation via down-regulating Akt/eNOS signaling

**Figure 5:**
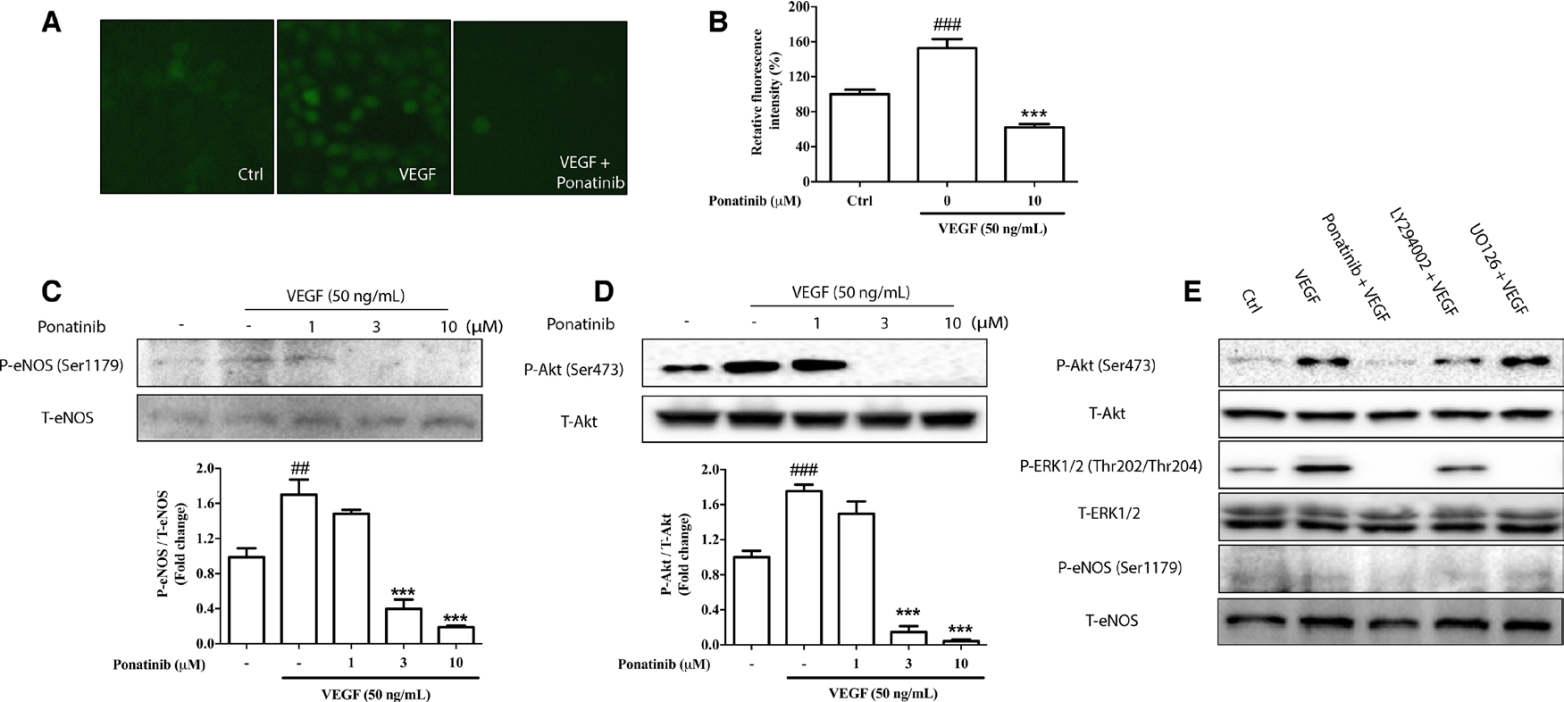
Effects of Ponatinib on VEGF-stimulated Akt/eNOS/NO pathway (**A**) Staining for NO production in HUVECs. HUVECs were treated with VEGF (50 ng/mL) in the absence or presence of 10 μM Ponatinib or 0.1% DMSO (vehicle control) for 12 h before staining with DAF-FM. (**B**) Quantification of the intracellular NO levels. (**C**) Effect of Ponatinib on VEGF-induced eNOS phosphorylation. (**D**) Effect of Ponatinib on VEGF-induced Akt phosphorylation at Ser473. HUVECs were treated with Ponatinib at indicated concentrations or 0.1% DMSO (vehicle control) and VEGF (50 ng/mL) for 1 h. The levels of eNOS or Akt phosphorylation were expressed as P-eNOS/T-eNOS or P-Akt/T-Akt (total) respectively. Values are presented as means ± SD (*n* = 3). ^###^*P* < 0.005 vs. control group; ^***^P < 0.005 vs. VEGF-treated group. (**E**) The effects of PI3K and ERK inhibitors on VEGF-induced eNOS phosphorylation. HUVECs were treated with VEGF (50 ng/mL) in the presence or absence of 10 μM Ponatinib, 10 μM LY294002 (PI3K inhibitor), or 10 μM U0126 (ERK inhibitor) for 1 h.

To evaluate the effect of Ponatinib on VEGF-mediated intracellular NO generation, the NO reactive probe, DAF-FM, was used to determine the intracellular NO level in HUVECs. As shown in Figure 5A and 5B, incubation of HUVECs with VEGF resulted in a significant increase in NO level to nearly 152% of the control after 3 h of incubation, whereas this increase was attenuated by treatment with Ponatinib. VEGF has been reported to increase NO generation by up-regulating the activity of eNOS via Akt signaling [35]. To confirm the result in HUVECs, we determined the level of phosphorylated eNOS in cultured cells by Western blotting (Figure 5C). VEGF significantly increased the level of eNOS phosphorylation, whereas Ponatinib suppressed the effect in a concentration-dependent manner. As the upstream activator of eNOS, Akt phosphorylation was also measured by Western blotting. As shown in Figure 5D, VEGF increased the level of phosphorylated Akt, whereas Ponatinib completely abolished both basal and VEGF-induced Akt phosphorylation at 3 μM. Further experiments showed that the phosphorylated Akt and eNOS were decreased by Ponatinib and PI3K inhibitor LY294002, whereas the ERK inhibitor UO126 did not show any effect on the Akt and eNOS phosphorylation (Figure 5E).

**Figure 6:**
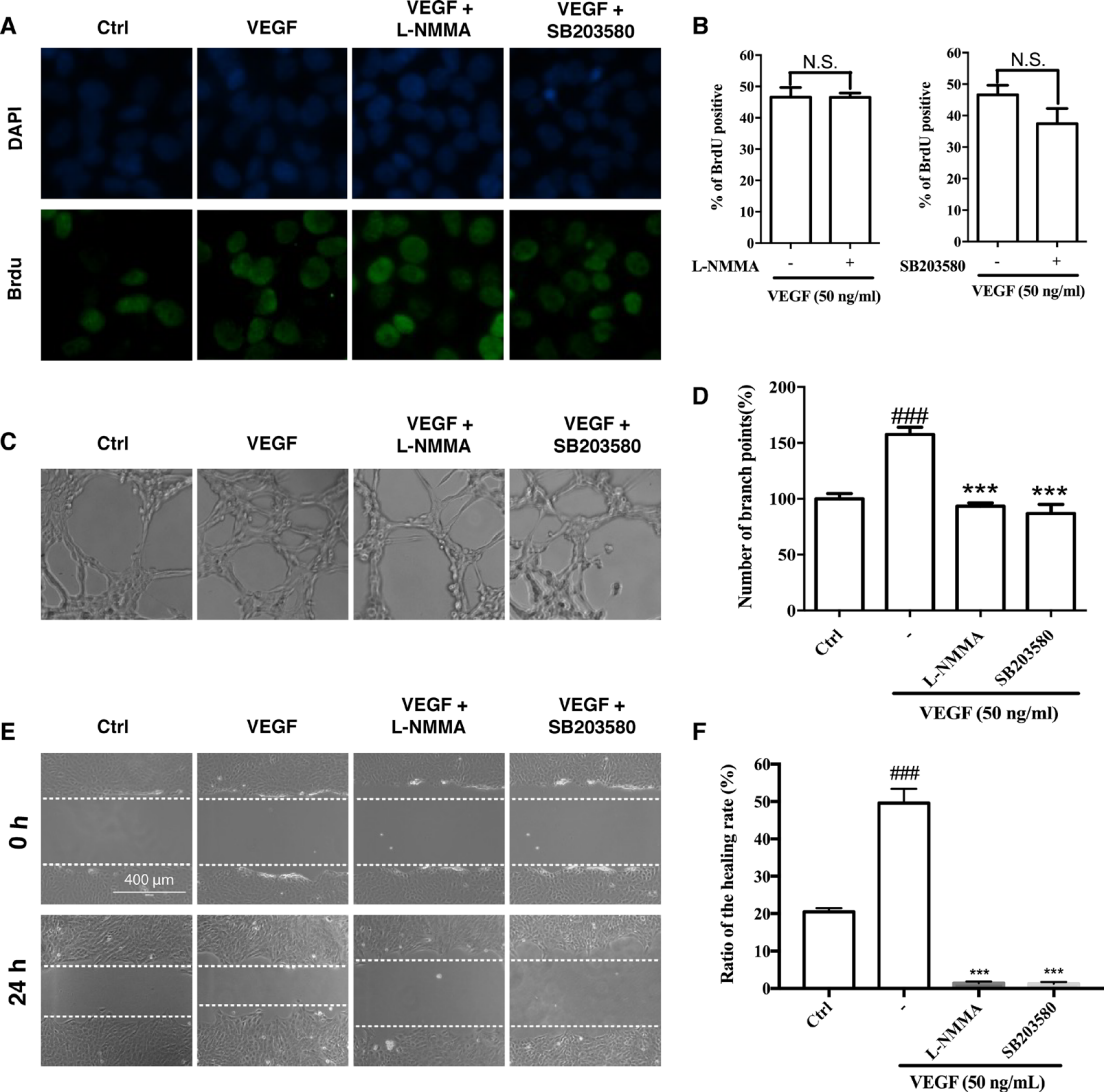
Effects of eNOS and p38 inhibitors on cell proliferation, migration and tube formation (**A** and **B**) Effects of eNOS and p38 inhibitors on VEGF-induced cell proliferation of HUVECs. The HUVECs were treated with VEGF in the presence or absence of L-NMMA (eNOS inhibitor; 200 μM) or SB203580 (p38 inhibitor; 10 μM) for 24 h. After immunostaining, the BrdU-positive cells were counted and presented as the percentage of all cells. (**C** and **D**) Effects of L-NMMA and SB203580 on VEGF-induced tube formation of HUVECs. The HUVECs were treated with VEGF alone or in combination with L-NMMA or SB203580 for 7 h. (**E** and **F**) Effects of L-NMMA and SB203580 on VEGF-induced migration of HUVECs in wound healing assay. The HUVECs were treated with VEGF in the presence or absence of L-NMMA or SB203580 for 24 h. Dashed lines indicate wound edges. The healing or closure rate was expressed as a ratio of the migration distance compared with the distance immediately after scratching. ^###^ *P* < 0.005 vs. control group; ^***^P < 0.005 vs. VEGF-treated group.

To evaluate the roles of eNOS in cell proliferation, migration and tube formation, we performed these assays in the presence of NOS inhibitor L-NMMA. As shown in Figure 6, L-NMMA had no effect on cell proliferation (Figure 6A and 6B), but it abolished both basal and VEGF-induced tube formation and migration (Figure 6C–6F).

### Ponatinib suppressed VEGF-induced ERK and p38 activation

**Figure 7:**
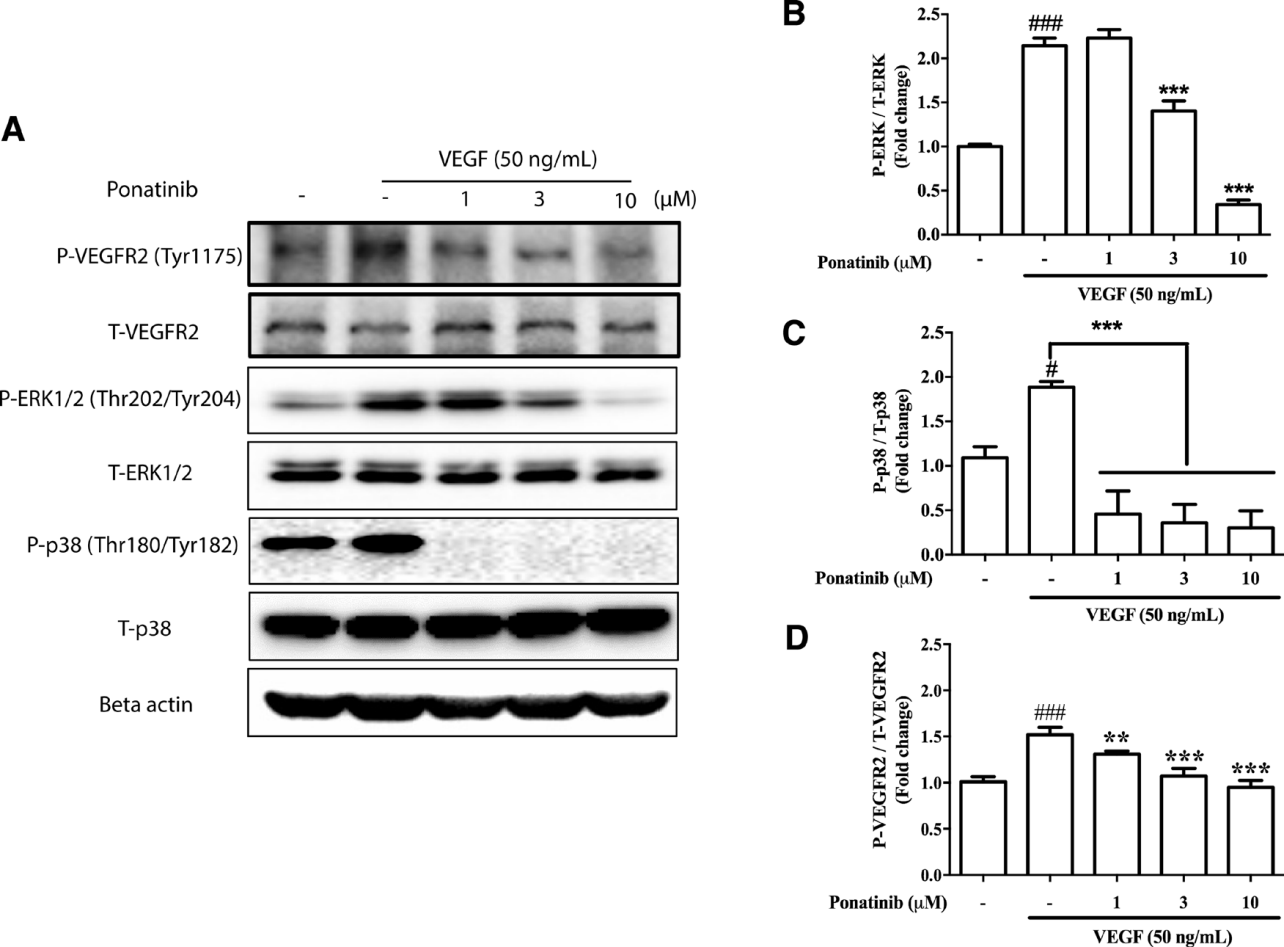
Effects of Ponatinib on VEGF-stimulated phosphorylation of VEGFR, ERK and p38 (**A**) Western blotting analysis for levels of phosphorylated VEGFR2, total VEGFR2, phosphorylated ERK1/2, total ERK1/2, phosphorylated p38, total p38 and beta-actin in HUVECs. The cells were treated with VEGF (50 ng/mL) in the presence or absence of Ponatinib at indicated concentrations or 0.1% DMSO (vehicle control) for 1 h followed by protein extraction. (**B**) Effect of Ponatinib on VEGF-induced ERK phosphorylation. (**C**) Effect of Ponatinib on VEGF-induced p38 phosphorylation. (**D**) Effect of Ponatinib on VEGF-induced VEGFR2 phosphorylation. The levels of ERK, p38 and VEGFR2 phosphorylation were expressed as P-ERK/T-ERK, P-p38/T-p38 and P-VEGFR2/T-VEGFR2 respectively. Values are presented as means ± SD (*n* = 3). ^#^*P* < 0.05 and ^###^*P* < 0.005 vs. control group; ^***^P < 0.005 vs. VEGF-treated group.

In addition to Akt, other kinases downstream of VEGF such as ERK and p38 also mediate endothelial cell proliferation and migration [36]. To determine if these kinases were involved in the anti-angiogenic effects of Ponatinib, the HUVECs were treated with VEGF and Ponatinib (1, 3, 10 μM) for 1 h followed by examination of ERK and p38 phosphorylation by Western blot analysis. Ponatinib significantly decreased the VEGF-induced phosphorylation of ERK and p38 in HUVECs in a concentration-dependent manner (Figure 7). Interestingly, the two pathways showed different sensitivity to Ponatinib inhibition. Ponatinib had no effect on VEGF-stimulated ERK phosphorylation at 1 μM and the effect became significant at 3 μM (Figure 7A and 7B); however, it completely abolished both basal and VEGF-induced p38 phosphorylation at all doses tested (Figure 7A and 7C). In addition to Akt, ERK and p38, Ponatinib also directly inhibited VEGF receptor (VEGFR). Over the concentration range tested (1-10 μM), it dose-dependently suppressed the phosphorylation of VEGFR2 (Figure 7A and 7D).

The blockade of p38 by Ponatinib was so drastic that we further examined its role in cell proliferation, migration and tube formation. SB203580, a p38 inhibitor, showed slight but not significant effect on cell proliferation (Figure 6A and 6B); however, it suppressed VEGF-induced tube formation (Figure 6C and 6D), and completely abolished both basal and VEGF-stimulated migration (Figure 6E and 6F).

## DISCUSSION

Angiogenesis is a hallmark of cancer development [2, 37]. To understand if anti-angiogenesis is involved in actions of clinically used anti-cancer drugs, we screened 114 FDA-approved anti-cancer drugs using the zebrafish model. Twelve drugs were found to have different levels of anti-angiogenic activity (Supplementary Table 1). This further demonstrates the value of the zebrafish model for investigating human diseases and drug screening [38–43]. In this study, we focused on Ponatinib for further investigation to understand how it works. We chose Ponatinib because its anti-angiogenic activity has not been reported, and there have been some case reports on its potential side effects of inducing blood clotting (thrombosis) and narrowing blood vessels [34]. We demonstrated that Ponatinib could significantly suppress angiogenesis in the zebrafish embryos. This effect was further confirmed by its inhibitory effects on basal and VEGF-induced activities in HUVECs in all assays related to angiogenesis, including BrdU assay on cell proliferation, transwell and wound healing assays on migration, and tube formation assay on vessel formation.

VEGF is a highly specific mitogen secreted by cells that stimulate angiogenesis. It stimulates angiogenesis-related cellular responses by binding to its tyrosine kinase receptors (VEGFR) on endothelial cell surface [44]. Cancer cells also secret VEGF, which is up-regulated by oncogenes, growth factors and hypoxia [45]. VEGFR is expressed at high levels in lymphatic endothelial cells, certain normal vessels, and tumor vessels [46]. Thus, the VEGF/VEGFR signaling pathway has become an attractive target for cancer therapy to block new blood vessel formation in growing tumors, resulting in tumor regression and suppression of metastasis [47, 48]. Our data showed clearly that Ponatinib could block all VEGF-induced cellular responses in cultured HUVECs, including phosphorylation of VEGFR2, PI3K/Akt, ERK and p38 pathways.

As a major intracellular signaling pathway involved in cell survival and migration, Akt mediates the signaling of a variety of cytokines and growth factors in a phosphatidylinositol-3 kinase (PI3K)-dependent manner [49]. In the endothelial cells, PI3K/Akt signaling pathway, which can be activated by VEGF, plays a critical role in a variety of cellular processes related to angiogenesis [50, 51]. PI3K/Akt has been reported to induce angiogenesis by activating endothelial nitric oxide (NO) synthase (eNOS) [52]. NO is a short-lived free radical product that mediates the effects of several key proangiogenic factors including VEGF, angiopoetin-2, and estrogen [53–55]. The enzyme eNOS, which catalyzes the generation of NO from L-arginine in blood vessels, is known to be involved in angiogenesis [56]. The bioactivity of eNOS is mainly determined by its localization and post-translational modifications such as phosphorylation [57]. Angiogenic factors induce phosphorylation of eNOS at Ser1179 via Akt signaling pathway, which provides a continuous flux of NO for inducing angiogenesis [52]. Thus, the regulation of eNOS/NO via phosphorylation/dephosphorylation events is one of key mechanisms in controlling angiogenesis and vessel function [58, 59]. In addition to eNOS, inducible NO synthase (iNOS)-induced NO signaling is also involved in the growth and survival of cancers through up-regulating tumor-secreted VEGF levels to induce angiogenesis [60, 61]. The significance of Akt/NOS/NO system in angiogenesis and cancer growth makes it a promising therapeutic target for cancer therapy.

In the present study, we demonstrated that the phosphorylation of eNOS, ERK, and Akt could all be suppressed by Ponatinib in a similar manner. However, only PI3K inhibitor LY294002 could reduce the phosphorylation of eNOS in VEGF-treated HUVECs, whereas ERK inhibitor UO126 did not seem to have this effect. This suggests that Ponatinib might reduce NO production by down-regulation of eNOS via Akt pathway. This agrees well with previous studies that the activity of eNOS was up-regulated by Akt following VEGF stimulation [62] and the inhibition of NO-related pathways could effectively reduce VEGF-induced endothelial cell migration and endothelial permeability [63]. The latter was confirmed in the present study because inhibition of NO pathway by NOS inhibitor L-NMMA suppressed VEGF-induced tube formation and abolished basal and VEGF-stimulated cell migration.

In addition to Akt/eNOS/NO pathway, VEGF/VEGFR also induces endothelial cell proliferation and migration via activation of other downstream kinases such as p38, ERK and FAK [64]. Our result showed that Ponatinib had powerful inhibitory effect on ERK and p38 activation, suggesting that blocking signal transduction of these pathways could also be part of the mechanism underlying the anti-angiogenic effect of Ponatinib. Interestingly, the three pathways including PI3K/Akt seemed to have different sensitivity to Ponatinib inhibition. Among the three, p38 was the most sensitive one as Ponatinib could completely abolish the basal and VEGF-stimulated p38 phosphorylation at 1 μM. Higher concentrations of Ponatinib were needed to exhibit the effects on Akt and ERK. Blockade of p38 with its inhibitor completely suppressed VEGF-induced tube formation and abolished basal and VEGF-induced cell migration, suggesting important roles for p38 in these cellular events.

The widespread targeting of all examined pathways downstream of VEGF/VEGFR suggests that Ponatinib may very likely target an upstream common point such as VEGFR. This view was supported by our evidence that Ponatinib suppressed VEGFR2 phosphorylation in response to VEGF. On the other hand, Ponatinib had differential effects on different downstream pathways, suggesting that the drug may also exert additional effects on specific pathways. This agrees well with the reports that Ponatinib is a multi-targeted kinase inhibitor which inhibits not only ABL kinase and its mutants, but also several upstream receptor tyrosine kinases including VEGFR, FGFR and PDGFR family members [27, 31, 33]. The inhibitory effects of Ponatinib on individual downstream signal transduction pathways have been reported in various cellular systems, including ERK in multiple CML cell lines [65] and endometrial cancer cells [33], Akt in murine myeloid cells [30] and endometrial cancer cells [33], and p38 MAPK in murine macrophages [66].

**Figure 8:**
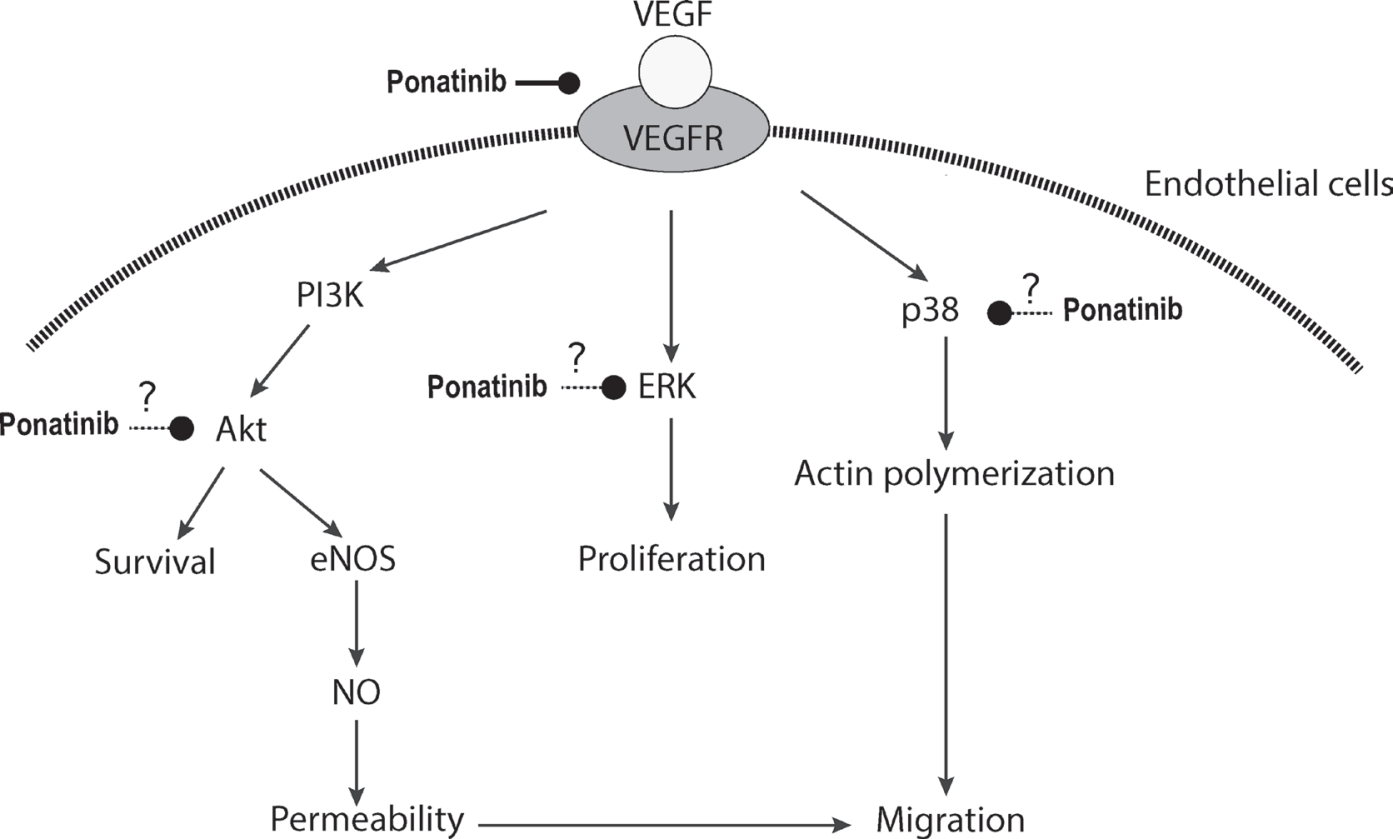
Schematic illustration of potential action mechanisms of Ponatinib in angiogenesis

In summary, our study demonstrated that Ponatinib was able to suppress angiogenesis in the zebrafish embryo *in vivo* and tube formation of HUVECs *in vitro*. Ponatinib inhibited all aspects of endothelial cell activity involved in angiogenesis including proliferation, permeability, migration and survival, and it likely exerted these effects by targeting VEGFR and its downstream signal transduction pathways including Akt/eNOS/NO and MAPKs (ERK and p38) (Figure 8). Our results in the present study provide further evidence for the anti-cancer effects of Ponatinib and its action mechanisms.

## MATERIALS AND METHODS

### Materials

Ponatinib was purchased from Selleckchem (Houston, TX). VEGFR tyrosine kinase inhibitor II (VRI) was purchased from Calbiochem (Darmstadt, Germany). 1-phenyl 2-thiourea (PTU) was obtained from Sigma-Aldrich (St. Louis, MO). 3-(4,5-dimethylthiazol-2-yl)-2,5-diphenyl tetrazolium bromide (MTT) and 4-amino-5-methylamino-2’,7’-difluorofluorescein (DAF-FM) were purchased from Molecular Probes (Eugene, OR). Medium 200 was purchased from Gibco Invitrogen (Carlsbad, CA). Primary antibodies for phospho-eNOS (Ser1179), total-eNOS, phospho-Akt (Ser473), total Akt, phospho-p38 (Thr180/Tyr182), total p38, phospho-ERK1/2 (Thr202/Tyr204), total ERK1/2, beta-actin and horseradish peroxidase-conjugated secondary antibodies were purchased from Cell Signaling Technology (Danvers, MA).

### Zebrafish care and maintenance

The *Tg*(*fli1a*:EGFP) transgenic zebrafish was used in the present study. The fish were maintained in the ZebTEC multilinking rack system (Tecniplast; Buguggiate, Italy) at 28°C on 14 h light:10 h dark photoperiod. For breeding, groups of male and female (3:2) adult zebrafish were placed in breeding boxes (Tecniplast) in the evening. The eggs were collected from the breeding boxes in the following morning, and normal and healthy fertilized eggs were selected under a stereomicroscope for treatment and observation.

### Treatment of zebrafish larvae

Zebrafish embryos at 2 hpf (hours after fertilization) were placed in a dish with 30 mL system water containing 0.1% 1-phenyl 2-thiourea (PTU). The egg shells were removed with forceps at 24 hpf, and the embryos were then transferred to a 24-well plate with 15 embryos and 1 mL system water per well. Three doses of Ponatinib (1, 3, and 10 μM) were tested for anti-angiogenic effect, and VRI (5 μM) and DMSO (0.1%) were used as positive and negative control respectively. For drug preparation, Ponatinib was first dissolved in DMSO to make a stock solution of 10 mM and it was diluted to the working concentrations before use. The embryos in the plate were treated with the drugs, and the ISVs and SIVs of the embryos were observed at 48 (24-h treatment) and 72 (48-h treatment) hpf respectively.

### Cell culture

HUVECs were purchased from the American Type Culture Collection (ATCC; Manassas, VA). The cells were cultured at 37°C in the Medium 200 supplemented with Low Serum Growth Supplement (LSGS) (Gibco) under humidified atmosphere with 5% CO_2_. The HUVEC cells of passage 3 to 7 were used in all experiments.

### MTT assay

The HUVECs were seeded into 96-well plates (5×10^3^ cells/well). After treatments, the cells were incubated in MTT solution (0.5 mg/mL) at 37°C for 4 h. The medium was then removed, and 100 μL DMSO was added to each well to dissolve the violet formazan crystals. Absorbance at 570 nm was measured by Infinite M200 PRO Multimode Microplate (Tecan; Männedorf, Switzerland). Cell viability was presented as a percentage compared with the control group.

### BrdU incorporation assay

Cell proliferation of HUVECs was determined by BrdU incorporation assay. HUVECs were seeded in 6-well culture plate and incubated with medium containing BrdU (3 μg/mL) and drugs for 24 h. The cells were then fixed with 4% paraformaldehyde and permeabilized with 0.1% Triton X-100 for immunostaining with BrdU antibody (Sigma). Images were visualized and photographed using the EVOS Cell Imaging System (Thermo Fisher; Waltham, MA). Each treatment was performed in triplicate and at least 200 nuclei in each sample were counted for BrdU-positive cells.

### Tube formation assay

The HUVECs (1 × 10^5^ cells) were incubated in 96-well plate with Ponatinib for 7–8 h in the presence or absence of VEGF (50 ng/mL) on a surface containing Matrigel (Corning; Corning, NY). Morphological changes of HUVECs and tube formation were visualized and photographed using the EVOS Cell Imaging System. The level of tube formation was quantified by counting the total number of branched tubes in each selected field. Each treatment was performed in triplicate and four fields were counted in each well to obtain an average (*n* = 3).

### Would healing assay

The HUVECs were seeded in 6-well plates and grown to confluence. A rectangular lesion was made in the monolayer with a cell scraper (IncuCyte; Essen BioScience, Ann Arbor, MI). Cells were rinsed three times with serum-free medium and incubated in the serum-free medium. After 24 h of incubation, three randomly selected fields at the lesion border were visualized and photographed using the EVOS Cell Imaging System. In each field, the distance between the edges of the lesion was determined and the healing rate was expressed as the ratio of the distance covered by the migrated cells compared with the distance at the beginning of the assay.

### Transwell migration assay

Cell migration assay was carried out using Costar transwell cell culture chambers (Corning). The lower and the upper chambers of each transwell were filled with Medium 200 (Thermo Fisher) containing no growth supplement. The HUVECs were exposed to the indicated concentrations of drugs in the medium in the upper transwell chamber. The bottom chamber contained medium with VEGF to serve as the chemo-attractant. After incubation at 37°C for 24 h, the cells on the upper side of the transwell membrane were removed using cotton swabs. The membrane and the migrated cells were then fixed in 4% paraformaldehyde in phosphate-buffered saline (PBS) for 15 min, and then stained with 1‰ DAPI for 15 min. The membrane was photographed on the EVOS Cell Imaging System, and the cells counted.

### Measurement of intracellular nitric oxide (NO)

The HUVECs were seeded into 6-well plates. After treatments, the cells were incubated in dark with the fluorescent probe, DAF-FM (5 μM), at 37°C for 15 min. The cells were then washed twice in PBS, and the fluorescence intensity was determined on the Infinite M200 PRO Multimode Microplate at an excitation wavelength of 495 nm and emission at 515 nm. The values of intracellular NO were normalized to the control group.

### Western blotting

Western blotting was performed as previously described [35]. Briefly, after treatments, the HUVECs were washed three times with ice-cold PBS, scraped with a scraper (SPL Life Sciences, Singapore) and collected into a tube. Then the harvested cells were lysed on ice for 30 min in RIPA lysis buffer containing 1% PMSF and 1% Protease Inhibitor Cocktail (Sigma-Aldrich), and centrifuged at 12,500 × g for 20 min at 4°C. The supernatant was collected and protein concentrations determined using the BCA protein assay kit (Thermo Scientific). Aliquots of protein samples (30 μg) were heated for 5 min at 95°C and electrophoresed on SDS-PAGE [10% (w/v) polyacrylamide gel] and then transferred to a polyvinylidene difluoride (PVDF) membrane (Bio-Rad; Hercules, CA). Subsequently, the membrane was blocked with 5% (w/v) non-fat milk in PBST (PBS containing 0.1% Tween-20) for 2 h at room temperature. The blots were incubated overnight at 4°C with primary antibodies. After washing with PBST for 20 min at room temperature, the membranes were further incubated with horseradish peroxidase-conjugated secondary antibodies for 2 h at room temperature. Finally, protein bands were visualized using ECL Plus Western Blotting Detection reagents (GE Healthcare; Piscataway, NJ). The membranes were then scanned on the ChemiDoc XRS Imaging System (Bio-Rad) and the intensity of the protein bands were analyzed using the Bio-Rad Image Lab Software (5.0).

### Statistical analysis

Statistical analysis was performed using Prism (GraphPad; San Diego, CA). All experiments were performed in triplicate. Data are expressed as means ± standard deviation (SD). Statistical analysis was carried out using one-way ANOVA followed by Tukey's multiple comparison, with *p* < 0.05 considered statistically significant.

## SUPPLEMENTARY MATERIALS FIGURES AND TABLES




