# Supplementary material for: Ponatinib exerts anti-angiogenic effects in the zebrafish and human umbilical vein endothelial cells via blocking VEGFR signaling pathway
Source: Oncotarget. 2018 Jan 10;9(62):31958–70. doi: 10.18632/oncotarget.24110 (PMC6112840; doi:10.18632/oncotarget.24110)
Supplement: Supplementary file 1 [file oncotarget-09-31958-s001.pdf]

Ponatinib exerts anti-angiogenic effects in the zebrafish and human umbilical vein endothelial cells via blocking VEGFR signaling pathway

SUPPLEMENTARY MATERIALS

Supplementary Table 1: Drugs screened in the zebrafish. See_Supplementary_Table 1
