# Supplementary material for: Ponatinib exerts anti-angiogenic effects in the zebrafish and human umbilical vein endothelial cells via blocking VEGFR signaling pathway
Source: Oncotarget. 2018 Jan 10;9(62):31958–70. doi: 10.18632/oncotarget.24110 (PMC6112840; doi:10.18632/oncotarget.24110)
Supplement: Supplementary file 2 [file oncotarget-09-31958-s002.docx]

**Supplementary Table 1: Drugs screened in the zebrafish**

| **NO.** | **Drug name** | **Anti-angiogenesis activity in zebrafish** |
| --- | --- | --- |
| 1 | 2-Methoxyestradiol | + |
| 2 | Apatinib | + |
| 3 | Axitinib | + |
| 4 | Cabozantinib | + |
| 5 | Camptothecin | + |
| 6 | Crizotinib | + |
| 7 | Ponatinib | + |
| 8 | Regorafenib | + |
| 9 | Sorafenib | + |
| 10 | Sunitinib | + |
| 11 | Topotecan HCl | + |
| 12 | Vandetanib | + |
| 13 | 10-DAB | - |
| 14 | Abitrexate | - |
| 15 | Adenine hydrochloride | - |
| 16 | Adrenalinium | - |
| 17 | Afatinib | - |
| 18 | Altretamine | - |
| 19 | Artemether | - |
| 20 | Aspirin | - |
| 21 | Atazanavir sulfate | - |
| 22 | Azacitidine | - |
| 23 | Azaguanine-8 | - |
| 24 | Azithromycin | - |
| 25 | Bendamustine HCl | - |
| 26 | Bepotastine Besilate | - |
| 27 | Bindarit | - |
| 28 | Bleomycin sulfate | - |
| 29 | Bortezomib | - |
| 30 | Bosutinib | - |
| 31 | Capecitabine | - |
| 32 | Carbazochrome sodium sulfonate | - |
| 33 | Carboplatin | - |
| 34 | Carmofur | - |
| 35 | Cephalomannine | - |
| 36 | Chlorpromazine | - |
| 37 | Cisplatin | - |
| 38 | Cladribine | - |
| 39 | Clofarabine | - |
| 40 | Clomifene citrate | - |
| 41 | Clorsulon | - |
| 42 | Cobicistat | - |
| 43 | Cortisone acetate | - |
| 44 | Cyclophosphamide monohydrate | - |
| 45 | Dacarbazine | - |
| 46 | DAPT | - |
| 47 | Dasatinib | - |
| 48 | Daunorubicin HCl | - |
| 49 | Diethylstilbestrol | - |
| 50 | Docetaxel | - |
| 51 | Doxorubicin | - |
| 52 | Eltrombopag | - |
| 53 | Epirubicin hydrochloride | - |
| 54 | Erlotinib HCl | - |
| 55 | Etoposide | - |
| 56 | Everolimus | - |
| 57 | Famciclovir | - |
| 58 | FK-506 | - |
| 59 | Floxuridine | - |
| 60 | Fludara | - |
| 61 | Fludarabine | - |
| 62 | Flunarizine 2HCl | - |
| 63 | Flutamide | - |
| 64 | Ftorafur | - |
| 65 | Fulvestrant | - |
| 66 | Gefitinib | - |
| 67 | Geniposidic acid | - |
| 68 | Genistein | - |
| 69 | Hydroxyurea | - |
| 70 | Ifosfamide | - |
| 71 | Irinotecan | - |
| 72 | Itraconazole | - |
| 73 | Lamotrigine | - |
| 74 | Laomustine | - |
| 75 | Lapatinib Ditosylate | - |
| 76 | Linagliptin | - |
| 77 | Lincocin | - |
| 78 | MDV3100 | - |
| 79 | Mercaptopurine | - |
| 80 | Mesna | - |
| 81 | Methazolastone | - |
| 82 | Mirabegron | - |
| 83 | Mitotane | - |
| 84 | Moroxydine | - |
| 85 | Nalocone HCl | - |
| 86 | Nelarabine | - |
| 87 | Nexium | - |
| 88 | Nilotinib | - |
| 89 | Nilvadipine | - |
| 90 | OSI-420 | - |
| 91 | Oxaliplatin | - |
| 92 | Paclitaxel | - |
| 93 | Paeoniflorin | - |
| 94 | Pazopanib HCl | - |
| 95 | Phenylbutazone | - |
| 96 | Physiomycine | - |
| 97 | Pimecrolimus | - |
| 98 | Pioglitazone | - |
| 99 | Pomalidomide | - |
| 100 | Rosiglitazone | - |
| 101 | Ruxolitinib | - |
| 102 | Streptozotocin | - |
| 103 | Sulindac | - |
| 104 | TAME | - |
| 105 | Temocapril HCl | - |
| 106 | Temsirolimus | - |
| 107 | Teniposide | - |
| 108 | Tofacitinib citrate | - |
| 109 | Tolnaftate | - |
| 110 | Tretinoin | - |
| 111 | Vemurafenib | - |
| 112 | Vincristine | - |
| 113 | Vismodegib | - |
| 114 | Vorinostat | - |
